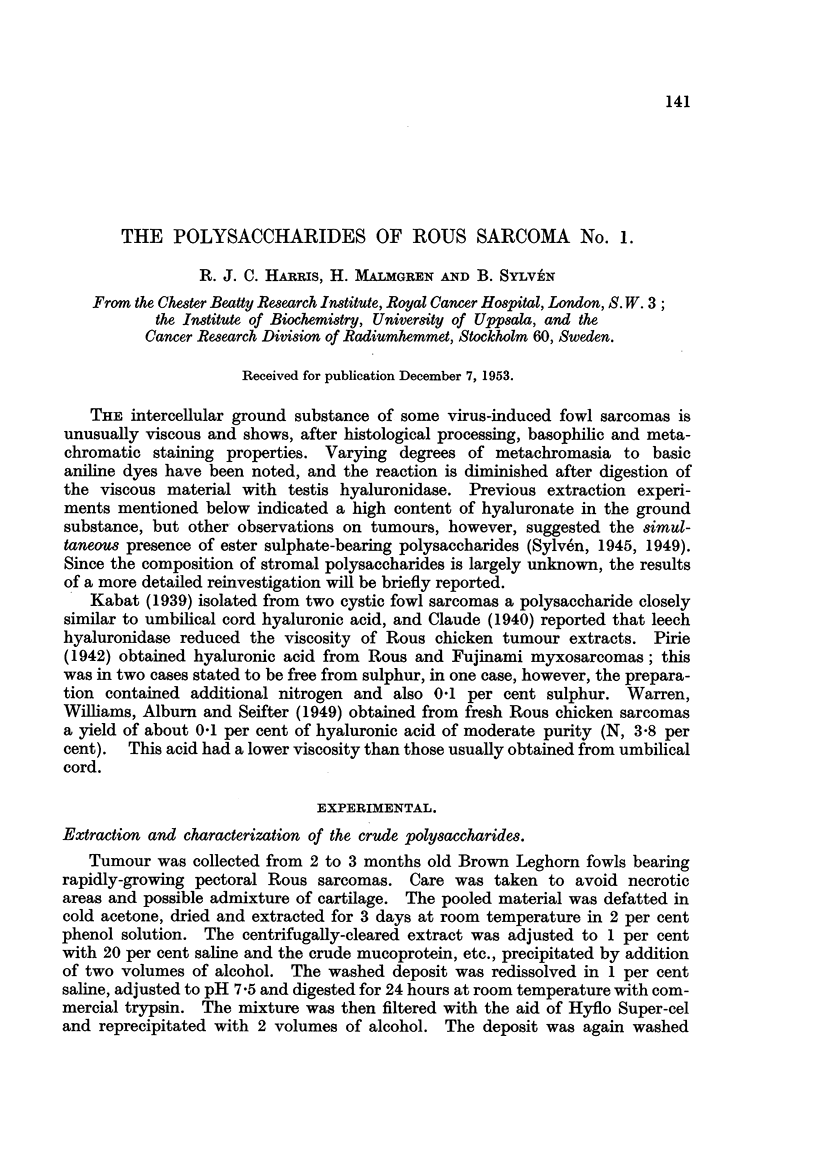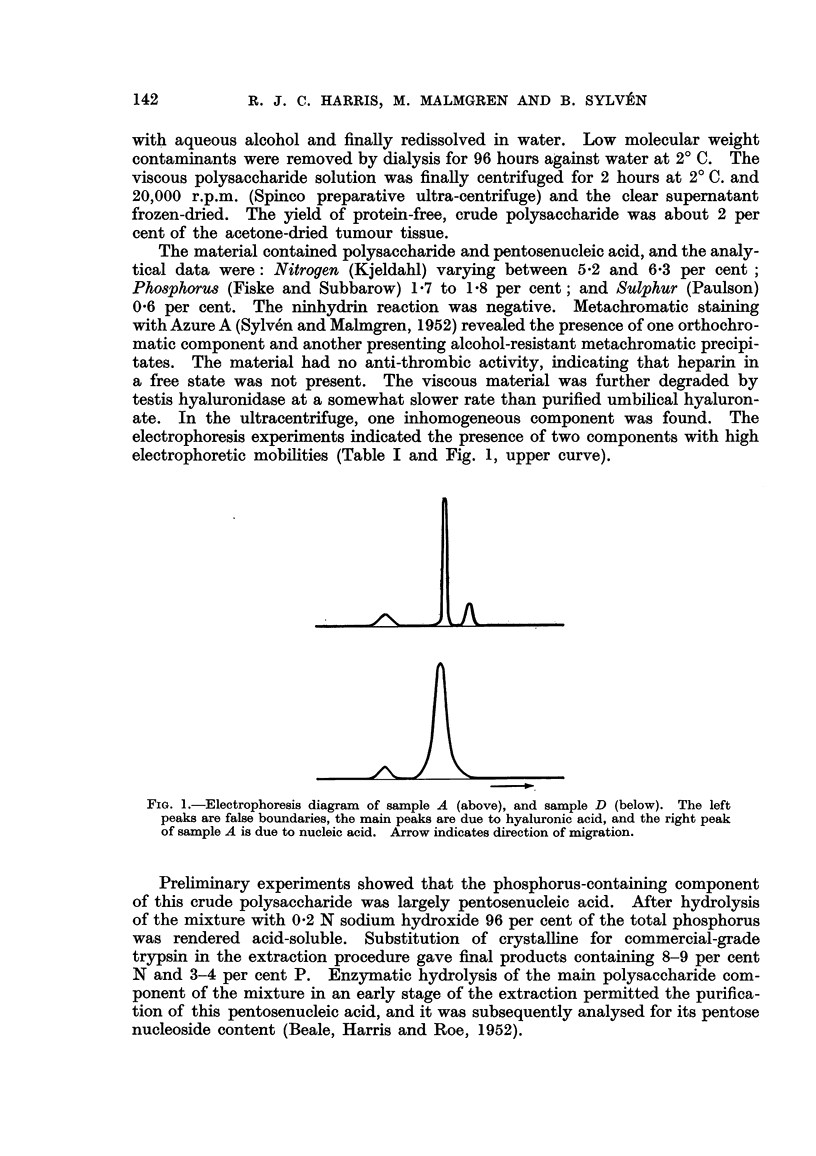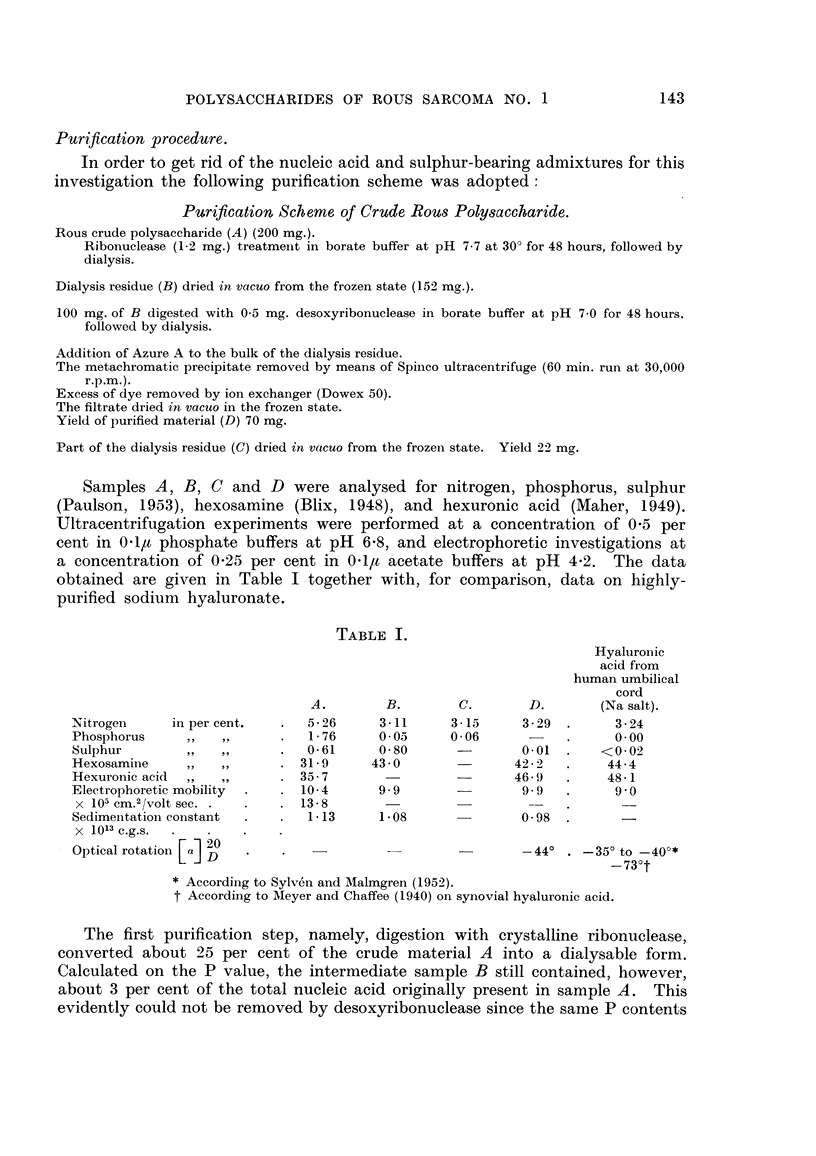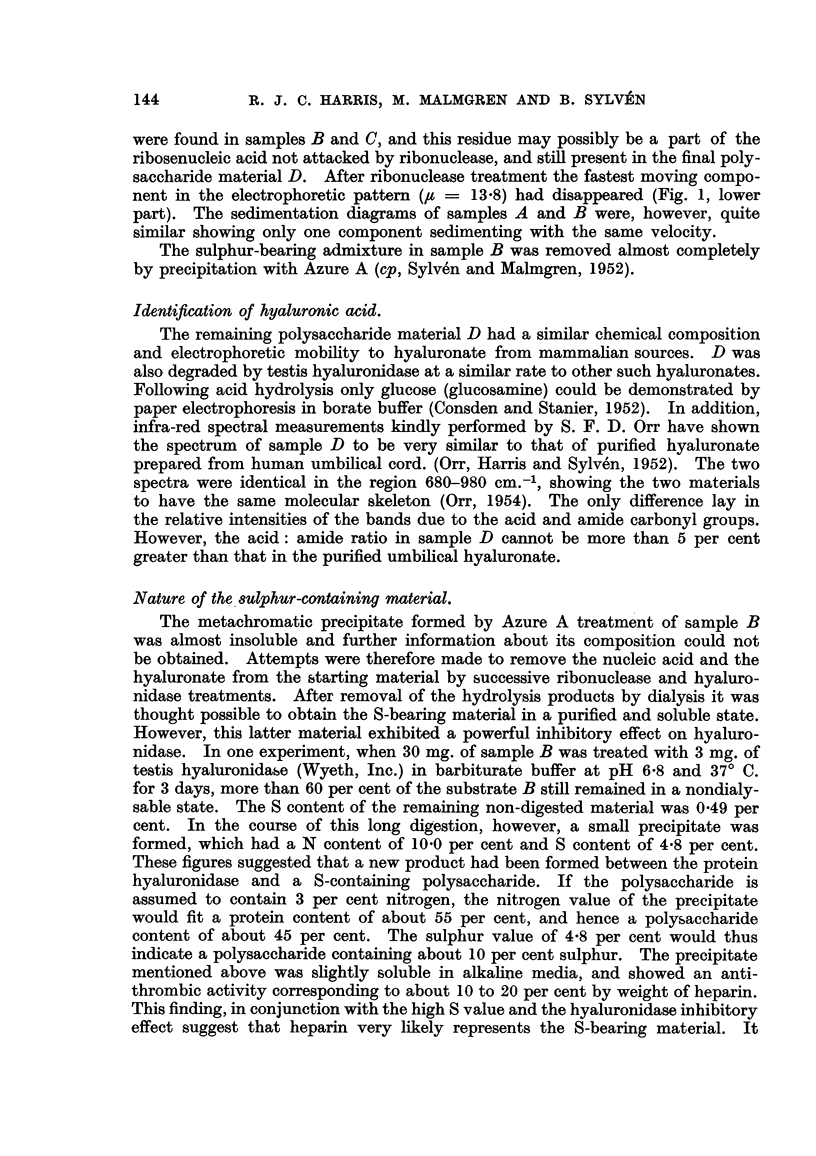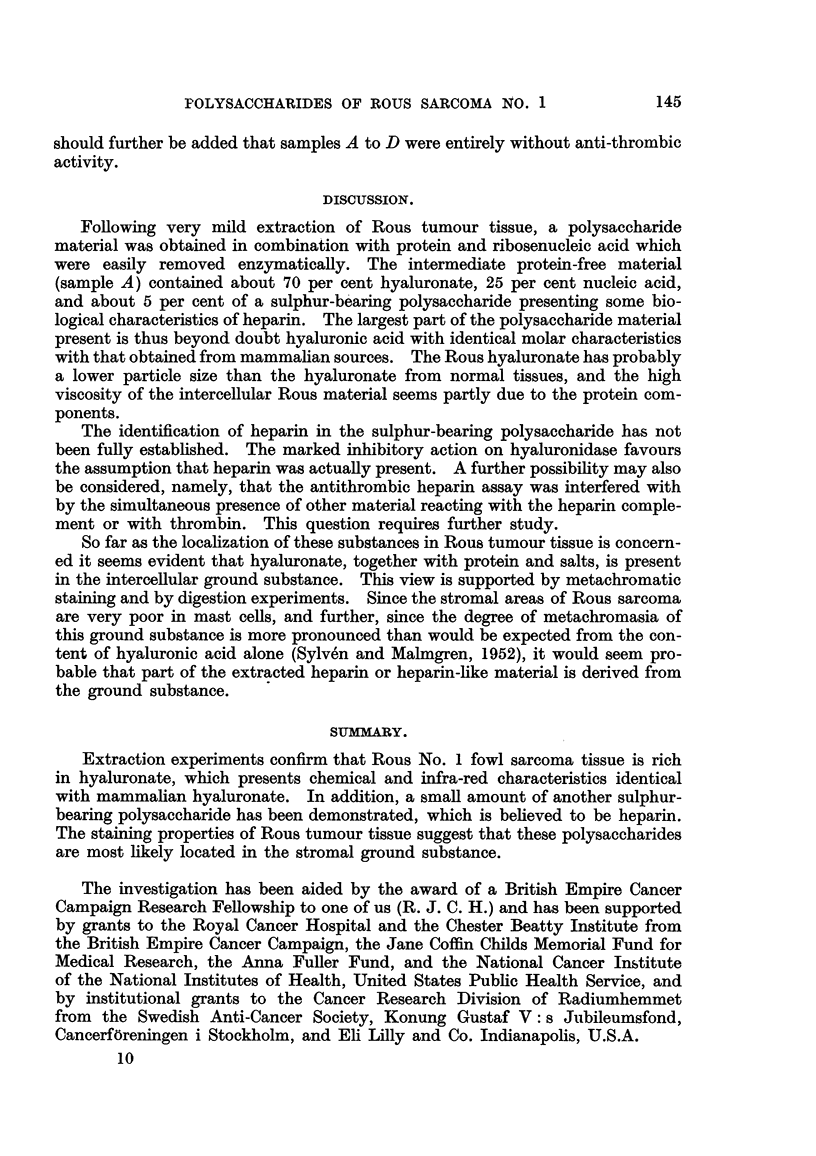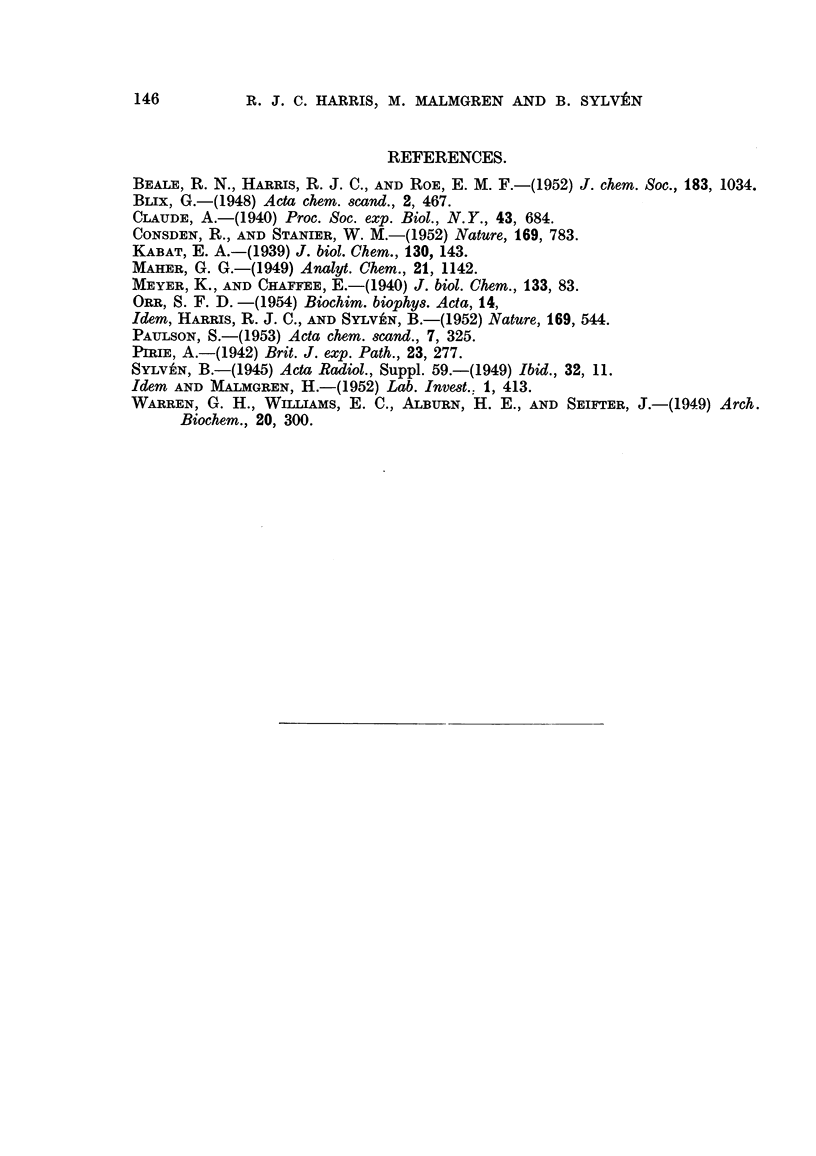# The Polysaccharides of Rous Sarcoma No. 1

**DOI:** 10.1038/bjc.1954.11

**Published:** 1954-03

**Authors:** R. J. C. Harris, H. Malmgren, B. Sylvén


### THE POLYSACCHARIDES OF ROUS SARCOMA No. 1.

R. J. C. HARRIS, H. MALMGREN AND B. SYLVÉN

*From the Chester Beatty Research Institute, Royal Cancer Hospital, London, S.W.* 3 ; *the Institute of Biochemistry, University of Uppsala, and the Cancer Research Division of Radiumhemmet, Stockholm* 60, *Sweden.*

Received for publication December 7, 1953.

THE intercellular ground substance of some virus-induced fowl sarcomas is unusually viscous and shows, after histological processing, basophilic and metachromatic staining properties. Varying degrees of metachromasia to basic aniline dyes have been noted, and the reaction is diminished after digestion of the viscous material with testis hyaluronidase. Previous extraction experiments mentioned below indicated a high content of hyaluronate in the ground substance, but other observations on tumours, however, suggested the *simultaneous* presence of ester sulphate-bearing polysaccharides (Sylvén, 1945, 1949). Since the composition of stromal polysaccharides is largely unknown, the results of a more detailed reinvestigation will be briefly reported.

Kabat (1939) isolated from two cystic fowl sarcomas a polysaccharide closely similar to umbilical cord hyaluronic acid, and Claude (1940) reported that leech hyaluronidase reduced the viscosity of Rous chicken tumour extracts. Pirie (1942) obtained hyaluronic acid from Rous and Fujinami myxosarcomas ; this was in two cases stated to be free from sulphur, in one case, however, the preparation contained additional nitrogen and also 0·1 per cent sulphur. Warren, Williams, Alburn and Seifter (1949) obtained from fresh Rous chicken sarcomas a yield of about 0·1 per cent of hyaluronic acid of moderate purity (N, 3·8 per cent). This acid had a lower viscosity than those usually obtained from umbilical cord.

### EXPERIMENTAL.

### *Extraction and characterization of the crude polysaccharides.*

Tumour was collected from 2 to 3 months old Brown Leghorn fowls bearing rapidly-growing pectoral Rous sarcomas. Care was taken to avoid necrotic areas and possible admixture of cartilage. The pooled material was defatted in cold acetone, dried and extracted for 3 days at room temperature in 2 per cent phenol solution. The centrifugally-cleared extract was adjusted to 1 per cent with 20 per cent saline and the crude mucoprotein, etc., precipitated by addition of two volumes of alcohol. The washed deposit was redissolved in 1 per cent saline, adjusted to pH 7·5 and digested for 24 hours at room temperature with commercial trypsin. The mixture was then filtered with the aid of Hyflo Super-cel and reprecipitated with 2 volumes of alcohol. The deposit was again washed with aqueous alcohol and finally redissolved in water. Low molecular weight contaminants were removed by dialysis for 96 hours against water at 2° C. The viscous polysaccharide solution was finally centrifuged for 2 hours at 2° C. and 20,000 r.p.m. (Spinco preparative ultra-centrifuge) and the clear supernatant frozen-dried. The yield of protein-free, crude polysaccharide was about 2 per cent of the acetone-dried tumour tissue.

The material contained polysaccharide and pentosenucleic acid, and the analytical data were: *Nitrogen* (Kjeldahl) varying between 5·2 and 6·3 per cent; *Phosphorus* (Fiske and Subbarow) 1·7 to 1·8 per cent; and *Sulphur* (Paulson) 0·6 per cent. The ninhydrin reaction was negative. Metachromatic staining with Azure A (Sylvén and Malmgren, 1952) revealed the presence of one orthochromatic component and another presenting alcohol-resistant metachromatic precipitates. The material had no anti-thrombic activity, indicating that heparin in a free state was not present. The viscous material was further degraded by testis hyaluronidase at a somewhat slower rate than purified umbilical hyaluronate. In the ultracentrifuge, one inhomogeneous component was found. The electrophoresis experiments indicated the presence of two components with high electrophoretic mobilities (Table I and Fig. 1, upper curve).

FIG. 1.—Electrophoresis diagram of sample *A* (above), and sample *D* (below). The left peaks are false boundaries, the main peaks are due to hyaluronic acid, and the right peak of sample *A* is due to nucleic acid. Arrow indicates direction of migration.

Preliminary experiments showed that the phosphorus-containing component of this crude polysaccharide was largely pentosenucleic acid. After hydrolysis of the mixture with 0·2 N sodium hydroxide 96 per cent of the total phosphorus was rendered acid-soluble. Substitution of crystalline for commercial-grade trypsin in the extraction procedure gave final products containing 8–9 per cent N and 3–4 per cent P. Enzymatic hydrolysis of the main polysaccharide component of the mixture in an early stage of the extraction permitted the purification of this pentosenucleic acid, and it was subsequently analysed for its pentose nucleoside content (Beale, Harris and Roe, 1952).

*Purification procedure.*

In order to get rid of the nucleic acid and sulphur-bearing admixtures for this investigation the following purification scheme was adopted :

*Purification Scheme of Crude Rous Polysaccharide.*

Rous crude polysaccharide (*A*) (200 mg.).
Ribonuclease (1·2 mg.) treatment in borate buffer at pH 7·7 at 30° for 48 hours, followed by dialysis.

Dialysis residue (*B*) dried *in vacuo* from the frozen state (152 mg.).

100 mg. of *B* digested with 0·5 mg. desoxyribonuclease in borate buffer at pH 7·0 for 48 hours, followed by dialysis.

Addition of Azure A to the bulk of the dialysis residue.
The metachromatic precipitate removed by means of Spinco ultracentrifuge (60 min. run at 30,000 r.p.m.).
Excess of dye removed by ion exchanger (Dowex 50).
The filtrate dried *in vacuo* in the frozen state.
Yield of purified material (*D*) 70 mg.

Part of the dialysis residue (*C*) dried *in vacuo* from the frozen state. Yield 22 mg.

Samples *A*, *B*, *C* and *D* were analysed for nitrogen, phosphorus, sulphur (Paulson, 1953), hexosamine (Blix, 1948), and hexuronic acid (Maher, 1949). Ultracentrifugation experiments were performed at a concentration of 0·5 per cent in 0·1$\mu$ phosphate buffers at pH 6·8, and electrophoretic investigations at a concentration of 0·25 per cent in 0·1$\mu$ acetate buffers at pH 4·2. The data obtained are given in Table I together with, for comparison, data on highly-purified sodium hyaluronate.

TABLE I.

| | *A*. | *B*. | *C*. | *D*. | Hyaluronic acid from human umbilical cord (Na salt). |
|---|---|---|---|---|---|
| Nitrogen in per cent. | 5·26 | 3·11 | 3·15 | 3·29 | 3·24 |
| Phosphorus ,, ,, | 1·76 | 0·05 | 0·06 | — | 0·00 |
| Sulphur ,, ,, | 0·61 | 0·80 | — | 0·01 | <0·02 |
| Hexosamine ,, ,, | 31·9 | 43·0 | — | 42·2 | 44·4 |
| Hexuronic acid ,, ,, | 35·7 | — | — | 46·9 | 48·1 |
| Electrophoretic mobility | 10·4 | 9·9 | — | 9·9 | 9·0 |
| $\times 10^5$ cm.$^2$/volt sec. | 13·8 | — | — | — | — |
| Sedimentation constant | 1·13 | 1·08 | — | 0·98 | — |
| $\times 10^{13}$ c.g.s. | | | | | |
| Optical rotation $[\alpha]_D^{20}$ | — | — | — | −44° | −35° to −40°* −73°† |

* According to Sylvén and Malmgren (1952).
† According to Meyer and Chaffee (1940) on synovial hyaluronic acid.

The first purification step, namely, digestion with crystalline ribonuclease, converted about 25 per cent of the crude material *A* into a dialysable form. Calculated on the P value, the intermediate sample *B* still contained, however, about 3 per cent of the total nucleic acid originally present in sample *A*. This evidently could not be removed by desoxyribonuclease since the same P contents were found in samples *B* and *C*, and this residue may possibly be a part of the ribosenucleic acid not attacked by ribonuclease, and still present in the final polysaccharide material *D*. After ribonuclease treatment the fastest moving component in the electrophoretic pattern ($\mu$ = 13·8) had disappeared (Fig. 1, lower part). The sedimentation diagrams of samples *A* and *B* were, however, quite similar showing only one component sedimenting with the same velocity.

The sulphur-bearing admixture in sample *B* was removed almost completely by precipitation with Azure A (*cp*, Sylvén and Malmgren, 1952).

*Identification of hyaluronic acid.*

The remaining polysaccharide material *D* had a similar chemical composition and electrophoretic mobility to hyaluronate from mammalian sources. *D* was also degraded by testis hyaluronidase at a similar rate to other such hyaluronates. Following acid hydrolysis only glucose (glucosamine) could be demonstrated by paper electrophoresis in borate buffer (Consden and Stanier, 1952). In addition, infra-red spectral measurements kindly performed by S. F. D. Orr have shown the spectrum of sample *D* to be very similar to that of purified hyaluronate prepared from human umbilical cord. (Orr, Harris and Sylvén, 1952). The two spectra were identical in the region 680–980 cm.$^{-1}$, showing the two materials to have the same molecular skeleton (Orr, 1954). The only difference lay in the relative intensities of the bands due to the acid and amide carbonyl groups. However, the acid : amide ratio in sample *D* cannot be more than 5 per cent greater than that in the purified umbilical hyaluronate.

*Nature of the sulphur-containing material.*

The metachromatic precipitate formed by Azure A treatment of sample *B* was almost insoluble and further information about its composition could not be obtained. Attempts were therefore made to remove the nucleic acid and the hyaluronate from the starting material by successive ribonuclease and hyaluronidase treatments. After removal of the hydrolysis products by dialysis it was thought possible to obtain the S-bearing material in a purified and soluble state. However, this latter material exhibited a powerful inhibitory effect on hyaluronidase. In one experiment, when 30 mg. of sample *B* was treated with 3 mg. of testis hyaluronidase (Wyeth, Inc.) in barbiturate buffer at pH 6·8 and 37° C. for 3 days, more than 60 per cent of the substrate *B* still remained in a nondialysable state. The S content of the remaining non-digested material was 0·49 per cent. In the course of this long digestion, however, a small precipitate was formed, which had a N content of 10·0 per cent and S content of 4·8 per cent. These figures suggested that a new product had been formed between the protein hyaluronidase and a S-containing polysaccharide. If the polysaccharide is assumed to contain 3 per cent nitrogen, the nitrogen value of the precipitate would fit a protein content of about 55 per cent, and hence a polysaccharide content of about 45 per cent. The sulphur value of 4·8 per cent would thus indicate a polysaccharide containing about 10 per cent sulphur. The precipitate mentioned above was slightly soluble in alkaline media, and showed an antithrombic activity corresponding to about 10 to 20 per cent by weight of heparin. This finding, in conjunction with the high S value and the hyaluronidase inhibitory effect suggest that heparin very likely represents the S-bearing material. It should further be added that samples *A* to *D* were entirely without anti-thrombic activity.

### DISCUSSION.

Following very mild extraction of Rous tumour tissue, a polysaccharide material was obtained in combination with protein and ribosenucleic acid which were easily removed enzymatically. The intermediate protein-free material (sample *A*) contained about 70 per cent hyaluronate, 25 per cent nucleic acid, and about 5 per cent of a sulphur-bearing polysaccharide presenting some biological characteristics of heparin. The largest part of the polysaccharide material present is thus beyond doubt hyaluronic acid with identical molar characteristics with that obtained from mammalian sources. The Rous hyaluronate has probably a lower particle size than the hyaluronate from normal tissues, and the high viscosity of the intercellular Rous material seems partly due to the protein components.

The identification of heparin in the sulphur-bearing polysaccharide has not been fully established. The marked inhibitory action on hyaluronidase favours the assumption that heparin was actually present. A further possibility may also be considered, namely, that the antithrombic heparin assay was interfered with by the simultaneous presence of other material reacting with the heparin complement or with thrombin. This question requires further study.

So far as the localization of these substances in Rous tumour tissue is concerned it seems evident that hyaluronate, together with protein and salts, is present in the intercellular ground substance. This view is supported by metachromatic staining and by digestion experiments. Since the stromal areas of Rous sarcoma are very poor in mast cells, and further, since the degree of metachromasia of this ground substance is more pronounced than would be expected from the content of hyaluronic acid alone (Sylvén and Malmgren, 1952), it would seem probable that part of the extracted heparin or heparin-like material is derived from the ground substance.

### SUMMARY.

Extraction experiments confirm that Rous No. 1 fowl sarcoma tissue is rich in hyaluronate, which presents chemical and infra-red characteristics identical with mammalian hyaluronate. In addition, a small amount of another sulphur-bearing polysaccharide has been demonstrated, which is believed to be heparin. The staining properties of Rous tumour tissue suggest that these polysaccharides are most likely located in the stromal ground substance.

The investigation has been aided by the award of a British Empire Cancer Campaign Research Fellowship to one of us (R. J. C. H.) and has been supported by grants to the Royal Cancer Hospital and the Chester Beatty Institute from the British Empire Cancer Campaign, the Jane Coffin Childs Memorial Fund for Medical Research, the Anna Fuller Fund, and the National Cancer Institute of the National Institutes of Health, United States Public Health Service, and by institutional grants to the Cancer Research Division of Radiumhemmet from the Swedish Anti-Cancer Society, Konung Gustaf V : s Jubileumsfond, Cancerföreningen i Stockholm, and Eli Lilly and Co. Indianapolis, U.S.A.

### REFERENCES.

BEALE, R. N., HARRIS, R. J. C., AND ROE, E. M. F.—(1952) *J. chem. Soc.*, **183**, 1034.
BLIX, G.—(1948) *Acta chem. scand.*, **2**, 467.
CLAUDE, A.—(1940) *Proc. Soc. exp. Biol., N.Y.*, **43**, 684.
CONSDEN, R., AND STANIER, W. M.—(1952) *Nature*, **169**, 783.
KABAT, E. A.—(1939) *J. biol. Chem.*, **130**, 143.
MAHER, G. G.—(1949) *Analyt. Chem.*, **21**, 1142.
MEYER, K., AND CHAFFEE, E.—(1940) *J. biol. Chem.*, **133**, 83.
ORR, S. F. D. —(1954) *Biochim. biophys. Acta*, **14**,
*Idem*, HARRIS, R. J. C., AND SYLVÉN, B.—(1952) *Nature*, **169**, 544.
PAULSON, S.—(1953) *Acta chem. scand.*, **7**, 325.
PIRIE, A.—(1942) *Brit. J. exp. Path.*, **23**, 277.
SYLVÉN, B.—(1945) *Acta Radiol.*, Suppl. 59.—(1949) *Ibid.*, **32**, 11.
*Idem* AND MALMGREN, H.—(1952) *Lab. Invest.*, **1**, 413.
WARREN, G. H., WILLIAMS, E. C., ALBURN, H. E., AND SEIFTER, J.—(1949) *Arch. Biochem.*, **20**, 300.